# Therapeutic Notes

**Published:** 1908-09

**Authors:** 


### Therapeutic Notes.

LA CLINIQUE, Paris, No. 32, May, 1907, calling attention to the fact that collargol would be discussed at the next Congress of Medicine in Paris, requested of its readers reports of their experiences with the remedy, whether good or bad. Of the responses received, all of which were favorable, the following are cited: Dr. Ruyssen had unguentum Credé inuncted repeatedly in a puerperal pyemia. Thereupon the lochias became copious and assumed the color of collargol solution, retaining it as long as unguentum Credé was used. Simultaneously with this phenomenon, which he ascribes to an excretion of collargol by the mucosæ of the inner genitals, a progressive retrogression of temperature and rapid recovery ensued. He also reports a dangerous erysipelas, cured with the ointment.

Professor Jeanbran has always seen success from a treatment of acute cystitis which he learned from Tavel in Berne, consisting of the daily injection of 10 to 20 cc. (2½ to 5 drams) of 1 to 3 per cent. collargol solution into the bladder. While silver nitrate injections are agonising, collargol causes no painful reaction whatever. Moreover, collargol more quickly inhibits the pain of the cystitic process and appears to have a more energetic action on the infectious cause. In chronic cystitis its action is less striking, as is the case with silver nitrate, since the causes (prostatic hypertrophy, and the like) continue. Here also collargol irrigations and injections are preferable, because of their painlessness. Dr. Frére has for some years used collargol in all acute infections to his satisfaction. He enumerates, as especially convincing, three cases—a grippe with pronounced meningeal symptoms, a double broncho-pneumonia, and an acute articular rheumatism; in these the sequence of the change in the clinical picture upon the intravenous collargol injection was particularly striking.

In a discussion of puerperal septicemia (*Amer. Jour. Obstet.*, June, 1907,) Dr. George T. Harrison, said: if one does not use collargol in these severe forms of infection, one has not done his duty. I have saved cases with it that I could not have cured in any other way. When intravenous injection can not be employed, collargol should be given by inunction or per rectum.

Dr. Charles S. Rockhill, Cincinnati, in the *Woman's Medical Journal,* March, 1908, on the Medical Treatment of Cholelithiasis," says it has been shown that a large portion of salicylic acid, given by mouth, passes into the bile, inhibits putrefactive processes and disinfects that secretion. Salicylic acid also has a powerful cholagogue action, producing a thin bile deficient in solids. It, therefore, seems to be ideal in its action, for it attacks the two most important etiological factors of gallstones,—infection and stagnation. Bauermeister has proposed a combination of salicylic acid, sodium oleate, menthol and phenolphthalein, the probilin pills. Dr. Rockhill has had experience therewith, and details the following case:

Mrs. P., aged 41, married. Had four children and was pregnant at the time I was called, June 21. I found her in great pain, temperature subnormal, with cold perspiration, some icterus, nausea and vomiting. Has had similar attacks.

I gave her a hypodermic injection of morphine, applied Priesnitz compresses, and washed out the stomach with hot Carslbad water. The symptoms subsided, and I discharged the patient with the usual instructions as to taking Carsbad salts, exercise, and the like. About two weeks later I was again called and found the same train of symptoms. In the mean time I had been advised to use probilin and concluded to try it in this case. After properly cleansing the bowels, I started with three pills morning and evening, increasing each dose one pill every second day until fourteen pills were taken daily for a period of three weeks. Then I gradually diminished the number, the treatment lasting three months. The patient had one attack, not as severe, shortly after she started the medication. Since then she has been well and free from all symptoms.

Dr. Rockhill has had three other cases that yielded in a similar manner, under the same treatment, as well as one patient who would not follow instructions, was operated upon and succumbed probably to a peritonitis.

Dr. Paul Cohnheim, Berlin, in his "Diseases of the Digestive Tract" (*Karger*, Berlin, 1908.) mentions, in the treatment of cholelithiasis, the use of probilin, three or four pills with one-third to one-half liter of very warm water twice daily, adding: "only when internal therapy has been exhausted, and when severe attacks occur again and again, should the patient be referred to the surgeon."

---

The Treatment of Cystitis.—Dr. Josef Bodenstein, Selzthal, (*Deut. Med. Presse*, No. 24, 1907,) reports that in 32 male and female cases of cystitis he gave arhovin (1 or 2 capsules 3 to 6 times daily) and an infusion of equal parts of herba herniariæ and herba chenopodium ambrosiadis (¼ to ¾ quart at each dose). Only in 4 of his 32 cases was the support of local treatment necessary. The results were brilliant; in 8 to 35 days most of the cases were cured or exhibited striking improvement. Within 1 to 3 days pain and tenesmus diminished and even urinary retention was favorably influenced. In 3 cases, catheterisation, previously required, could be discontinued after a few days of arhovin medication. Urine with thick sediment of pus and mucus sometimes became entirely clear within 8 to 14 days; with the amelioration of the bladder difficulties the fever often disappeared on the second or third day; appetite improved and intestinal irregularity ceased without any other measures. The effect, curative and analgesic, was astonishing because of its promptness, certainty and intensity.

Dr. M. A. Fechheimer, of Detroit, writing on "The Treatment of Chronic Gonorrhea," (*Detroit Medical Journal,* March, 1908), says: "Arhovin is useful in those cases where there appears to be an irritation of the prostatic urethra, and acts in a very beneficial manner."

---

HEMORRHOIDS.

Zweig, of Vienna, says in the chapter on hemorrhoids of his "Therapie der Magen und Darmkrankheiten" (Urban & Schwarzenberg, 1907): "The anusol suppositories have been found very useful by me; they are a compound of bismuth with iodised resorcin-sulphonicacid, and are introduced daily after the morning defecation and at night before retiring."

---

EXODIN.

George M. Gould's "American Year-book of Medicine and Surgery," 1905, brings in the chapter of materia medica an abstract of Ebstein's report on exodin, according to which it stands midway between the laxatives and purgatives and produces mushy stools without interfering with the digestion or causing colic. The editors of the department, R. W. Wilcox and A. A. Stevens, add. "We have found this substance to fulfill well the claims made for it."

*Chronic Constipation in Children.*—H. B. Sheffield, M. D., Instructor in Diseases of Children and Attending Pediatrist (O.P.D.) Post-Graduate Medical School and Hospital; Visiting Pediatrist to the Metropolitan Hospital and Dispensary, etc., New York, writes in a paper (*Archives of Pediatrics,* Nov., 1905): "Of the more recently recommended cathartics, I found exodin to act splendidly. It is tasteless and, therefore, readily taken by the little patients, either in apple sauce, syrup of acacia or in tablets. Three to 10 grains produce a soft movement without any gastrointestinal disturbance."

In *American Medicine* of May 6, 1905, the department of "Treatment," edited by Drs. S. Solis Cohen, L. F. Appleman and F. Lindauer, comments on exodin as follows: "This is a most satisfactory laxative for palliative use in cases of chronic constipation until massage and electricity have established normal peristalsis without drugs. It gives soft stools without straining. The dose is from 0.5 gm. to 1.5 gm. once or twice daily"

In discussing purgation before and after abdominal operations, H. J. Boldt, said: "I have for a couple of years resorted to giving in very many instances a cathartic perhaps half an hour before operation, and, as a rule, would have the desired effect within 24 hours. The cathartic which has given more satisfaction than any other is a preparation called exodin, which seems to have a better effect in this way: it does not cause griping and produces normal evacuation in from 20 to 24 hours after administration. Then we have the effect following the operation."—*Trans. So. Surg. and Gyn. Assn.*, 1904.

In Professor Schwalbe's *Jahrbuch der Praktischen Medicin*, 1905, Prof. M. Cloetta, of Leipzig, reviews the reports that have been published on exodin and expresses his own opinion on the remedy as follows: "It is a fact that the use of exodin is not attended by any symptoms of irritation; the passages follow without disturbances of any kind and are in the nature of normal evacuations.

---

PYRENOL IN PERTUSSIS AND ASTHMA.—Eulenburg's "Encyclopädische Jahrbücher der gesamten Heilkunde," 1906, says regarding pyrenol: "It continues to demonstrate its trustworthiness, especially in bronchial asthma and pertussis. It prevents pulmonary complications and loosens expectoration, as for instance in bronchial catarrh. In angina pectoris it exercises a sedative effect. It can be used in phthisis cases, but it may be well to avoid it if hemoptysis threatens."

---

PYRENOL IN CROUPOUS PNEUMONIA.—Keiner (*Münch. Med. Woch.*, July 24, 1906.) says that in the Dortmund Municipal Hospital pyrenol was found to be the most effective of all the remedies for croupous pneumonia. Cases admitted on the third and fourth days of the disease ran a prompt course when pyrenol in 7½-grain doses was given every two or three hours. He has been favorably impressed by its therapeutic efficacy and is convinced of its pertinency and harmlessness.

---

UROTROPIN IN CONVALESCENCE FROM TYPHOID.—Frederick C. Shattuck, Jackson Professor of Clinical Medicine in Harvard University, says in a paper on "The Value of Drugs in Therapeutics (*Bost. Med. and Surg. Journ.*, March 29, 1906,) that some useful synthetic compounds have been discovered and more will be discovered. Who can count the number of cases of typhoid which have originated through the urine of a previous patient? This is a danger against which urotropin, rightly used, seems to afford absolute protection. For the past eight years his patients have all had 7 to 10 grains of urotropin thrice daily, two successive days of each week, until convalescence was complete. In no case has harm resulted. Were the practice universal, there would be less typhoid, the prevalence of which is a reproach to our civilisation.

